# ‘Single-shot’ pulmonary vein isolation using a novel lotos pulsed field ablation catheter: a pre-clinical evaluation of feasibility, safety, and 30-day efficacy

**DOI:** 10.1093/europace/euad362

**Published:** 2023-12-18

**Authors:** Sijia Pu, Fangzhou Liu, Yuhan Chen, Cihua Luo, Peng Li, Yanlin Chen, Lu Fu, Huiyi Liu, Xingdong Ye, Shulin Wu, Yumei Xue, Weidong Lin

**Affiliations:** School of Medicine, South China University of Technology, Guangzhou, China; Guangdong Cardiovascular Institute, Guangdong Provincial People’s Hospital (Guangdong Academy of Medical Sciences), Southern Medical University, 106 Zhongshan Er Rd, Guangzhou 510080, China; Guangdong Cardiovascular Institute, Guangdong Provincial People’s Hospital (Guangdong Academy of Medical Sciences), Southern Medical University, 106 Zhongshan Er Rd, Guangzhou 510080, China; Guangdong Cardiovascular Institute, Guangdong Provincial People’s Hospital (Guangdong Academy of Medical Sciences), Southern Medical University, 106 Zhongshan Er Rd, Guangzhou 510080, China; Insight Medtech Co., Ltd, Shenzhen, China; Insight Medtech Co., Ltd, Shenzhen, China; Guangdong Cardiovascular Institute, Guangdong Provincial People’s Hospital (Guangdong Academy of Medical Sciences), Southern Medical University, 106 Zhongshan Er Rd, Guangzhou 510080, China; Guangdong Cardiovascular Institute, Guangdong Provincial People’s Hospital (Guangdong Academy of Medical Sciences), Southern Medical University, 106 Zhongshan Er Rd, Guangzhou 510080, China; Guangdong Cardiovascular Institute, Guangdong Provincial People’s Hospital (Guangdong Academy of Medical Sciences), Southern Medical University, 106 Zhongshan Er Rd, Guangzhou 510080, China; Guangdong Cardiovascular Institute, Guangdong Provincial People’s Hospital (Guangdong Academy of Medical Sciences), Southern Medical University, 106 Zhongshan Er Rd, Guangzhou 510080, China; Guangdong Cardiovascular Institute, Guangdong Provincial People’s Hospital (Guangdong Academy of Medical Sciences), Southern Medical University, 106 Zhongshan Er Rd, Guangzhou 510080, China; School of Medicine, South China University of Technology, Guangzhou, China; Guangdong Cardiovascular Institute, Guangdong Provincial People’s Hospital (Guangdong Academy of Medical Sciences), Southern Medical University, 106 Zhongshan Er Rd, Guangzhou 510080, China; Guangdong Cardiovascular Institute, Guangdong Provincial People’s Hospital (Guangdong Academy of Medical Sciences), Southern Medical University, 106 Zhongshan Er Rd, Guangzhou 510080, China

**Keywords:** Catheter ablation, Pulsed field ablation, Atrial fibrillation, Pulmonary vein isolation, Single shot, Nanosecond

## Abstract

**Aims:**

Pulsed field ablation (PFA) is emerging as a non-thermal, tissue-specific technique for pulmonary vein isolation (PVI) in atrial fibrillation therapy. This pre-clinical study aims to investigate the feasibility and safety of PVI using a novel PFA system including a nanosecond-scale PFA generator, a novel lotos PFA catheter, and a customized 12 Fr steerable sheath.

**Methods and results:**

A total of 11 Yorkshire swine were included in this study, with 4 in the acute cohort and 7 in the chronic cohort. Under general anaesthesia, transseptal puncture and pulmonary vein (PV) angiography was initially performed. The PFA catheter was navigated to position at the right and left PV antrum after the electroanatomic reconstruction of the left atrium. Biphasic PFA applications were performed on PVs in both the spindle-shaped and the lotos-shaped poses. Pulmonary vein isolation and PFA-associated safety were assessed 30 min after ablation in both cohorts and 30 days later in the chronic cohort. Detailed necropsy and histopathology were performed. Additional intracardiac echocardiography and coronary angiogram were evaluated for safety. All target PVs (*n* = 20) were successfully isolated on the first attempt. No spasm of coronary artery or microbubble was seen during the procedure. Eleven of 12 PVs (91.6%) remained in isolation at the 30-day invasive study. No evidence of PV stenosis was observed in any targets. However, transient diaphragm capture occurred in 17.6%. Histopathological examinations showed no evidence of collateral injury.

**Conclusion:**

This study provides scientific evidence demonstrating the safety and efficacy of the novel PFA catheter and system for single-shot PVI, which shows great potential.

## Graphical Abstract

**Graphical Abstract:**
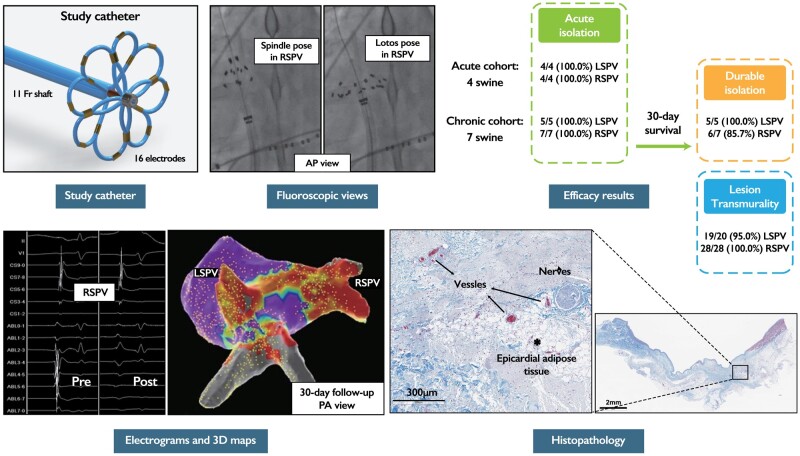

**What’s new?**

- This pre-clinical study demonstrated a novel lotos catheter for single-shot pulmonary vein isolation that achieved 100% acute isolation and 91.6% 30-day efficacy with acceptable safety. Histological examination suggests transmural lesion.
- The catheter is designed with eight splines, and each of the two adjacent splines is linked with electrodes, creating a stable lotos configuration that makes it easy for the catheter to reach the targets even with sharp turns. Moreover, the structure eliminates the need for rotation for additional ablation, simplifying the workflow.
- The catheter design, which includes two typical poses, allows it to be easily fitted to a variety of pulmonary vein antrum sizes.
- The application of nanosecond-scale pulses minimizes the risk of capturing the phrenic nerves, which could result in minimal or no pain in clinical scenarios.

## Introduction

Catheter ablation is an established treatment for atrial fibrillation (AF) as recommended in current guidelines, and pulmonary vein isolation (PVI) is the cornerstone.^1–4^ However, the currently used classic ablation techniques, radiofrequency (RF) and cryoballoon (CB) ablation, cannot meet the clinical demands of cardiomyocyte-specific damage.^5^ Non-selective damage of vascular smooth muscle cells has long been a shadow of thermal energy ablation, making pulmonary vein (PV) stenosis a serious complication following traditional cardiac ablation.^6,7^ Additionally, adjacent anatomical structures such as the ganglionated plexus or the oesophagus may be affected during the procedure, resulting in abnormal cardiac autonomic regulation and/or oesophageal injury.^5,8^

Pulsed field ablation (PFA), with its safety and efficacy demonstrated in numerous publications,^9–14^ has shown PFA as a potential alternative in the future. It can selectively target the atrial myocardium, while sparing adjacent tissues due to the lower irreversible electroporation (IRE) threshold specific to cardiomyocytes compared with other types of cells.^15–17^ Moreover, it has been proved that approaches using ‘single-shot’ circumferential PVI (CPVI) are more attractive than point-by-point approaches due to their simpler workflow and shorter procedure time.^5,18^ Thus, a single-shot PFA catheter holds great promise for AF therapy, given its feasibility for CPVI and its specific feature of causing cardiomyocyte-selective damage. It is recognized, however, that the operating convenience and the energy control of current PFA catheters are quite inadequate. Despite this, standardizing the assessment of PFA catheters from one to another and determining the optimal universal pulse parameter remains a huge challenge, given the different types of energy delivery among these PFA catheters, which are influenced by pulse parameters as well as catheter and electrode design.^19–22^ Furthermore, different catheter designs and pulse parameter settings can impact the catheter’s contact with tissue and the number of recommended applications (e.g. whether the rotation is required for additional applications). It also plays an essential role in interfering with collateral tissues (e.g. capturing the phrenic nerve).^19,23^ Consequently, outcomes in previous assessments of current PFA catheters are not duplicable in terms of effectiveness and safety for new products.

In this pre-clinical study, we evaluated the feasibility and safety of a novel PFA system. The system includes a nanosecond-scale pulsed field generator, an 11 Fr over-the-wire expandable lotos catheter for single-shot PVI, and a customized 12 Fr steerable sheath. The catheter design and the nanosecond-scale energy delivery parameters simplify the manipulation and minimize the risk of capturing the phrenic nerve during the procedure. The effectiveness of the system was evaluated by measuring PV potentials, PV bidirectional blocking, and doing electroanatomic mapping before, immediately after, and 30 days after ablation. Gross pathology, histopathology, as well as surrounding tissue injury, are assessed simultaneously.

## Methods

### Animals and protocols

All the experiments were performed in Guangdong Jinshi Medical Technology Service Co., Ltd and were approved by the Institutional Animal Care and Use Committee (IACUC) of the Guangdong Jinshi Medical Technology Service Co., Ltd (approval number IACUC-T-2022-013). All procedures followed the Guideline for the Care and Use of Laboratory Animals of Guangdong Provincial People’s Hospital. A total of 11 Yorkshire swine were included in this study, with 4 in the acute cohort and 7 in the chronic cohort.

### Pulsed field ablation system and catheter

**Figure 1:**
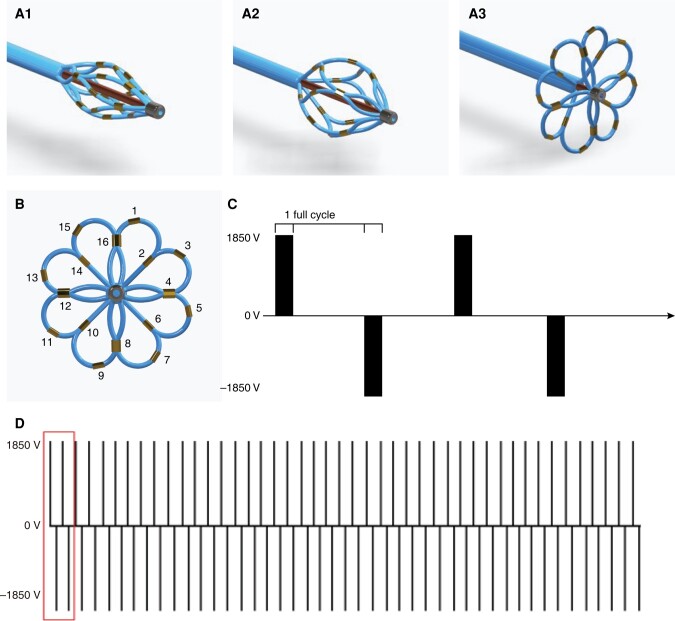
The study catheter and the pulse parameter used for bipolar PFA. (*A1–A3*) Multi-electrode catheter gradually deploying into a basic pose (*A1*), a spindle pose (*A2*), and a lotos pose (*A3*). with an 11 Fr shaft. (*B*) Details of the fully deployed lotos pose (the distal portion is 28 or 31 mm in diameter), shown with 16 electrodes in total. (*C* and *D*) Details of a biphasic pulses set. A full cycle consists of a pair of diphasic pulses. PFA, pulsed field ablation.

The ablation system in this study comprised a PFA generator (InRythm, Insight Lifetech, Shenzhen, China), a proprietary lotos-shaped PFA Catheter (LotosPFA, Insight Lifetech), and a customized 12 Fr steerable sheath (InBridge, Insight Lifetech) to navigate and position the PFA catheter. The PFA generator is capable of delivering high-voltage pulsed field waveforms over multiple channels with variable amplitudes. The 11 Fr over-the-wire PFA ablation catheter with 16 electrodes was arranged in a lotos configuration when being fully deployed, with a transition state as a spindle (*Figure 1A1–A3*). As shown in *Figure 1B*, each electrode was assigned a specific number. Biphasic waves are generated by electrode pairs consisting of one odd-numbered electrode and one even-numbered electrode. All 16 electrodes were capable of recording bipolar electrograms, as well as pacing. The catheter tip was advanced over a 0.035 J-tipped guidewire to reach the PV ostia in its spindle shape and achieve optimal contact with the PV antrum with its lotos framework. The catheter was available in two sizes, with tip diameters of 28 and 31 mm in lotos pose, respectively. This allows for positioning the catheter tip at the PV antrum effectively and achieving appropriate contact, regardless of anatomic variations.

Trains of nanosecond-scale pulses were delivered in a bipolar, biphasic fashion in each electrode pair (*Figure 1D*). All electrode pairs were employed in sequence during one delivery process. Arcing and muscular motion were avoided during the delivery of PFA by using a proprietary optimized catheter and programme design including electrode size, spacing, pulse width, and voltage amplitude.

Pulsed field ablation waveforms consist of a sequence of nanosecond-scale pulses, with an amplitude of 1850 V for the spindle pose and 2100 V for the lotos pose. The delivery procedure for each vein contains two to four applications in the spindle pose and additional two to four applications in the lotos pose, which depends on the operator’s judgement. The total application duration is <1 min for one target.

### Procedural details

The swine ablation model was described previously.^19^ In brief, 4-month-old Yorkshire swine (50–60 kg) were administered general anaesthesia with mechanical ventilation. Rivaroxaban (150 mg orally every day) was administered for 7 days before PFA. A constant irrigation of the PFA catheter was maintained throughout the procedure to prevent thrombosis. The procedures were conducted under heparin anticoagulation with an activated clotting time range of 300–350 s.

A coronary sinus multi-electrode catheter was placed via left femoral access for sensing and pacing. A single transeptal puncture was performed with the 12 Fr custom-steerable sheath (InBridge, Insight Lifetech) for left atrial access by using a standard transeptal technique. Pulmonary venous angiography was performed to obtain baseline PV diameter. HD Grid (Abbott, Chicago, IL, USA) was used for activation and voltage mapping of the left atrium (LA) with the EnSite™ NavX™ Precision Three-dimensional mapping system (Abbott).

The PFA catheter was navigated into the LA and the wire tip was positioned within a distal PV branch. The catheter tip was advanced into the right superior PVs (RSPVs) and left superior PVs (LSPVs) and then expanded gradually by operating the actuator in the handle under fluoroscopic guidance. Tissue contact was judged only by resistance during catheter push manoeuvres and trains of nanosecond-scale biphasic pulses were delivered in the spindle shape in the PV ostia and then in the lotos shape in the PV antra. There is no requirement for these pulses to be synchronized with either atrial or ventricular depolarization.

For immediate and 30-day efficacy assessment, high-density voltage maps (including the assessment of vein potentials) were prepared using the HD Grid catheter after the 30 min waiting period and 30 days later, along with a PV bidirectional block test.

For safety assessment, venous angiography was performed to assess the diameter of each vein before and after ablation. In addition, intracardiac echocardiography (ICE) was used to inspect the presence of microbubbles generated during ablation. A coronary angiogram was performed simultaneously with ablation in several swine in order to evaluate the impact of PFA on coronary arteries. Once all assessments were completed, the study catheter was removed and examined for thrombus or tissue attachment, and ICE was conducted to inspect the presence of iatrogenic pericardial effusion.

### Follow-up and histological investigation

All acute cohort swine were humanely sacrificed on the same day after completing all procedures. All swine in the chronic cohort were recovered and monitored for 30 days. A repeat procedure including electroanatomic mapping, PV potential recording, PV bidirectional block test, and PV angiography was conducted after 30 days. Both cohorts of swine were euthanized, and their hearts and adjacent tissues were collected for gross pathological and histopathological analyses at the end of their predetermined survival periods.

Upon documentation of any abnormalities or injuries, all explanted hearts and neighbouring organs were fixed in formalin. The ablated PVs were identified, opened along their long axes, and trimmed evenly along the vein axis to obtain four circumferential longitudinal sections per PV. Samples were embedded in paraffin, sectioned into slides, and stained with Haematoxylin and Eosin and Masson’s Trichrome.

A veterinary pathologist blinded to the electroanatomic data and outcomes assessed the slides. The sections of veins with myocardial sleeves were carefully assessed for the presence and transmurality of lesions, extent of fibrosis, damage to nerves, arterioles, and venules within the lesion, presence of oedema, level of inflammatory response, presence of thrombi or haemorrhage, and any other relevant findings.

The oesophagus and other adjacent tissues were carefully examined for gross pathology and photographed. Since no abnormalities were visible on the surface, no area of interest was sectioned for histological analysis.

### Statistical analyses

Continuous variables were presented as mean ± standard deviation (SD), whereas categorical variables were presented as count and percentage. A two-sided Student’s *t*-test was employed to compare continuous variables between two groups. In the case of comparisons among more than two groups, one-way analysis of variance (ANOVA) analysis and the Tukey multiple comparisons test were used, as appropriate. Categorical variables were compared using *χ*^2^ analysis or Fisher’s exact test, as appropriate. A *P* value <0.05 was considered statistically significant. Statistical analyses were performed with SPSS 24.0 software (SPSS Inc., Chicago, IL, USA), and graphs were generated using GraphPad Prism software (version 9.1.1).

## Results

### Procedural outcomes

**Figure 2:**
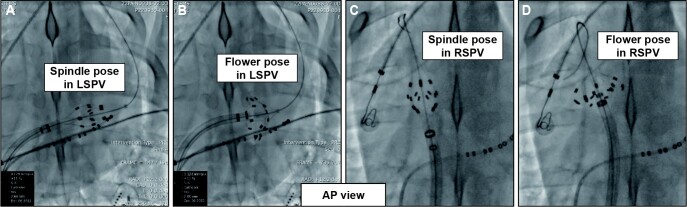
Fluoroscopic views. (*A* and *B*) PFA catheter deployed over-the-wire in LSPVs of swine in the spindle (*A*) and the lotos (*B*) pose through a deflectable sheath. Sensing and pacing catheters were placed in the coronary sinus. (*C* and *D*) A PFA catheter deployed over-the-wire in RSPVs of swine in the spindle (*C*) and the lotos (*D*) pose through a deflectable sheath. Sensing and pacing catheters were placed in the coronary sinus. LSPV, left superior pulmonary vein; PFA, pulsed field ablation; RSPV, right superior pulmonary vein.

The study catheter was successfully navigated into all targets with the spindle pose at the PV ostia and the lotos pose at the PV antrum, as confirmed by the fluoroscope (*Figure 2*). Pulsed field ablation was delivered to all targeted PVs, with an average of 3.7 ± 0.8 applications in the spindle pose and 3.5 ± 0.9 applications in the lotos pose. The number of applications for each PV is shown in Supplementary material online, *Table S1*. Post-ablation and 30-day follow-up assessments were successfully performed.

### Efficacy assessment

#### Acute cohorts

**Figure 3:**
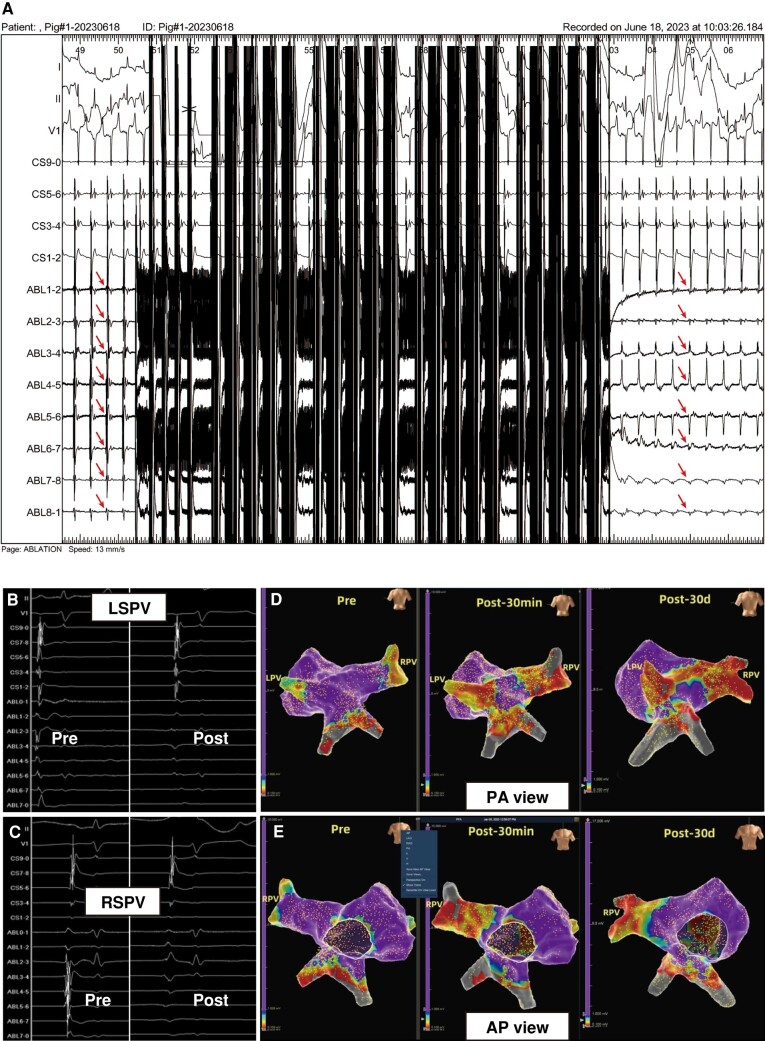
Representative electrograms and electroanatomic maps. (*A*) PV potential disappearing immediately following ablation. (*B* and *C*) Changes of pulmonary vein electrograms obtained from the study catheter before and immediately after PFA in the LSPV (*B*) and RSPV (*C*), (*D* and *E*), Representative voltage maps performed at baseline, immediately after ablation, and 30-day follow-up showing the extent and chronic lesion of isolation in the LSPV and RSPV. LSPV, left superior pulmonary vein; PFA, pulsed field ablation; RSPV, right superior pulmonary vein.

In four swine, all targeted PVs (four RSPVs and four LSPVs) were successfully isolated at the first attempt (*Table 1*, *Figure 3A–C*). Electroanatomic maps showed low-voltage areas (<0.1 mV) when the target PVs were remapped after a 30 min waiting period. In general, acute PVI was achieved in 100% of PVs.

**Table 1:** Procedural and histologic features in the chronic and acute cohorts of PFA

	Chronic (*N* = 12)			Acute (*N* = 8)		
	LSPV (*n* = 5)	RSPV (*n* = 7)	All (*N* = 12)	LSPV (*n* = 4)	RSPV (*n* = 4)	All (*N* = 8)
Catheter pose when discharging	Spindle; lotos	Spindle; lotos	NA	Spindle; lotos	Spindle; lotos	NA
Acute isolation	5/5 (100.0)	7/7 (100.0)	12/12 (100.0)	4/4 (100.0)	4/4 (100.0)	8/8 (100.0)
Chronic isolation	5/5 (100.0)	6/7 (85.7)	11/12 (91.6)	NA	NA	NA
Phrenic palsy	0/5 (0.0)	0/7 (0.0)	0/12 (0.0)	0/4 (0.0)	0/4 (0.0)	0/8 (0.0)
PV stenosis (immediately)	0/5 (0.0)	0/7 (0.0)	0/12 (0.0)	0/4 (0.0)	0/4 (0.0)	0/8 (0.0)
** **PV diameter, pre vs. post, *P*-value	8.1 ± 0.3 vs. 8.2 ± 0.4, *P* = 0.9733^a^	13.5 ± 1.0 vs. 13.1 ± 1.4, *P* = 2.200^a^		8.2 ± 1.3 vs. 8.2 ± 1.4, *P* > 0.9999^a^	14.0 ± 2.1 vs. 13.9 ± 2.5, *P* = 0.7211^a^	
30-day follow-up						
** **Phrenic palsy	0/5 (0.0)	0/7 (0.0)	0/12 (0.0)	NA	NA	NA
** **Cerebral embolism	0/5 (0.0)	0/7 (0.0)	0/12 (0.0)	NA	NA	NA
** **PV narrowing (>70%)	0/5 (0.0)	0/7 (0.0)	0/12 (0.0)	NA	NA	NA
** **PV diameter, pre vs. 30 days, *P*-value	8.1 ± 0.3 vs. 8.2 ± 0.4, *P* = 1.060^b^	13.5 ± 1.0 vs. 13.4 ± 0.8, *P* = 0.2267^b^		NA	NA	NA
	**LSPV (*n* = 20)**	**RSPV (*n* = 28)**	**All (*n* = 48)**			
Histology						
** **Pulmonary vein						
** **Lesion thickness, mm	0.82 ± 0.54	1.32 ± 0.60	1.11 ± 0.62			
** **Transmurality	19/20 (95.0)	28/28 (100.0)	47/48 (97.9)			
** **Homogenous lesion	20/20 (100.0)	28/28 (100.0)	48/48 (100.0)			
** **Endocardial sparing	20/20 (100.0)	28/28 (100.0)	48/48 (100.0)			
** **Skeleton retention/structural framework preserved	20/20 (100.0)	28/28 (100.0)	48/48 (100.0)			
** **Thrombus	0/20 (0.0)	0/28 (0.0)	0/48 (0.0)			
** **Arteriolar, venular, and nerve damage	0/20 (0.0)	0/28 (0.0)	0/48 (0.0)			

PV stenosis refers to PV diameter decreasing >70%. ‘Pre’ means pre-ablation; ‘post’ means immediately post ablation; 30 days means at 30-day follow-up. Values are mean ± SD, *n*/*N* (%).

NA, not applicable; LSPV, left superior pulmonary vein; PFA, pulsed field ablation; PV, pulmonary vein; RSPV, right superior pulmonary vein.

^a^Delivered by Student’s *t*-test.

^b^Delivered by the Tukey multiple comparisons test.

#### Chronic cohorts

The LSPVs of two swine were excluded due to the difficulties of catheter assessment because of the low-voltage areas of the PV antra shown in baseline electroanatomic maps. Similar to acute cohorts, all targeted PVs, including seven RSPVs and five LSPVs, achieved acute isolation, which was assessed by PV potential recording, bidirectional block test, and electroanatomic mapping at baseline and after the 30 min waiting period (*Figure 3A–E*). All swine completed a follow-up period averaging 32.1 ± 2.0 days. In the 30-day follow-up, reassessment revealed short-term isolation in five out of five LSPVs (100%) and six out of seven RSPVs (85.7%; *Table 1*). Overall, the 30-day efficacy rate of PVI in this cohort was 91.6%.

### Safety assessment

**Figure 4:**
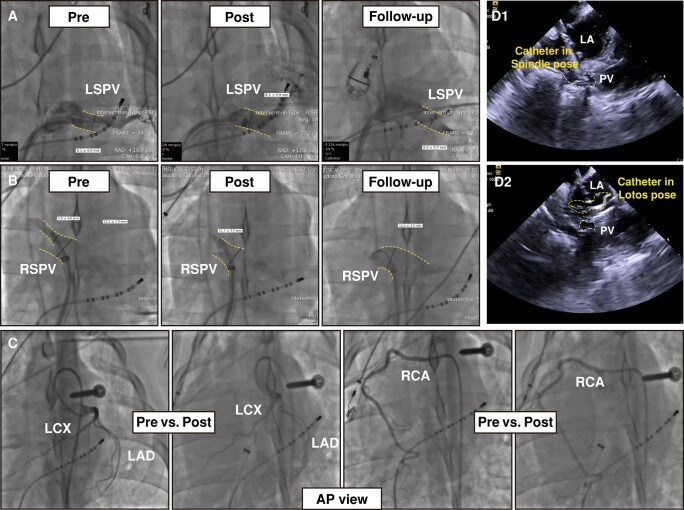
Fluoroscopic views and intracardiac echocardiographic views for safety assessment. (*A* and *B*) Baseline, immediately post ablation, and 30-day follow-up venous angiography of the LSPV (*A*) and RSPV (*B*) showing no venous stenosis. The dotted line denotes pulmonary veins. (*C*) A view of the coronary artery before and 8 min after ablation. (*D*) Intracardiac echocardiographic view of PVs during ablation with spindle pose (D1) and lotos pose (D2), showing no microbubbles. LSPV, left superior pulmonary vein; RSPV, right superior pulmonary vein.

No catheter placement or deployment-associated complications occurred during the procedure (e.g. pericardial effusion, cardiac tamponade, phrenic palsy, or evidence of an air embolism). No significant difference in PV diameter between the pre-ablation and 30 min post-ablation groups was observed. Similar results were found in the 30-day follow-up group compared with the pre-ablation group (*Table 2*, *Figure 4A* and *B*).

**Table 2:** Measurements of pulmonary vein diameters at different points of time

	Pre (mm)	Post (mm)	30 days (mm)	*P*-value	Pre vs. post^c^	Pre vs. 30 days^c^	Post vs. 30 days^c^
Acute cohort							
LSPV (*n* = 4)	8.2 ± 1.3	8.2 ± 1.4	NA	>0.9999^a^	NA	NA	NA
RSPV (*n* = 4)	14.0 ± 2.1	13.9 ± 2.5	NA	0.7211^a^	NA	NA	NA
Chronic cohort							
LSPV (*n* = 5)	8.1 ± 0.3	8.2 ± 0.4	8.2 ± 0.4	0.6561^b^	0.7826	0.7502	0.9664
RSPV (*n* = 7)	13.5 ± 1.0	13.1 ± 1.4	13.4 ± 0.8	0.3432^b^	0.3331	0.9860	0.6349

‘Pre’ means pre-ablation; ‘post’ means 30-minute post ablation; 30 days means at 30-day follow-up. Values are mean ± SD.

ANOVA, analysis of variance; LSPV, left superior pulmonary vein; RSPV, right superior pulmonary vein.

^a^Delivered by a paired Student’s *t*-test.

^b^Delivered by one-way ANOVA.

^c^Delivered by the Tukey multiple comparisons test.

During PFA deliveries, neither phrenic palsy, notable arrhythmias, nor ST-segment elevations occurred. The coronary angiography was performed concurrently with PFA in two swine, and no evidence of coronary spasm was observed (*Figure 4C*). No microbubbles were detected by ICE during PFA (*Figure 4D*, see Supplementary material online, *Video S1*). Neither symptoms of phrenic palsy nor cerebral embolism were detected during the follow-up period.

However, transient phrenic nerve capture was observed in several cases, especially when the PFA catheter was expanded in the spindle pose (*Table 3*). In general, phrenic nerve capture occurred in 29.4% of PFA applications in the spindle pose, and 5.9% in the lotos pose, resulting in a total of 17.6%. Phrenic nerve capture was more frequently observed in the LSPV than in the RSPV (27.3 vs. 8.6%).

**Table 3:** Events of phrenic nerve capture in different poses

	Spindle pose	lotos pose	Total	*P*-value^a^
LSPV	8/16 (50.0)	1/17 (5.9)	9/33 (27.3)	0.0045*
RSPV	2/18 (11.1)	1/17 (5.9)	3/35 (8.6)	0.5808
All	10/34 (29.4)	2/34 (5.9)	12/68 (17.6)	0.0109*

Values are *n*/*N* (%). Comparison is made between PVs that were subjected to ablation in the spindle and flower pose.

LSPV, left superior pulmonary vein; RSPV, right superior pulmonary vein.

^a^Delivered by Fisher’s exact test.

^*^ *P* < 0.05.

### Histological investigation

**Figure 5:**
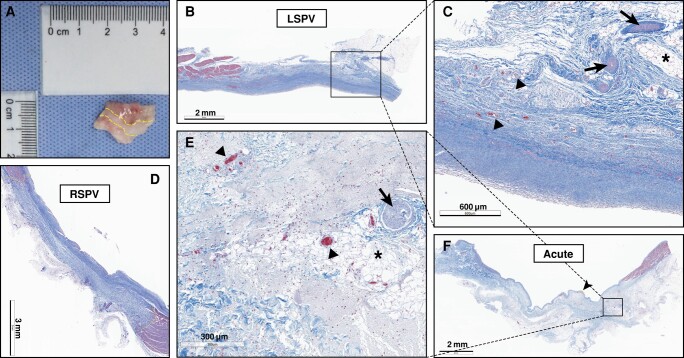
Gross pathology and histopathology of PFA lesions. (*A*) A PFA lesion in the LSPV with the contiguous broad lesion, shown in gross appearance prior to formalin fixation. The lesion can be identified as white areas of discolouration and is outlined with the dotted line. (*B–D*) Representative histopathologic images of the chronic cohort of transmural PFA lesion in the LSPV (*B* and *C*) and RSPV (*D*) with structural framework preserved and endocardial sparing, which was conducted 30 days after the procedure. Haemorrhage and myocardium inflammation can be seen. (*C*) A higher magnification image of the black box outlined in *B*, demonstrating a sparing of the arteries, veins (arrowhead), and nerves (arrow). (*E* and *F*) Representative histopathologic images of target PV in the acute cohort, demonstrating oedema and mild neoendocardium (arrowhead in *F*). (*E*) A close-up of the black box area in (*F*), showing the preserved structural framework with vessels (black arrowhead) and nerves (arrow) retention. The black asterisk represents unaffected epicardial adipose tissue. Masson trichrome staining was used in (*B–F*). LSPV, left superior pulmonary vein; PFA, pulsed field ablation; RSPV, right superior pulmonary vein.

The entire hearts and adjacent tissues of all swine were successfully extracted, showing macroscopic lesion formation at each target area in each animal. On the endocardial surface, lesions were identified as white patches (*Figure 5A*). No ablation-related macroscopic lesions were observed in neighbouring organs, including phrenic nerves and oesophagus (see Supplementary material online, *Figure S1*).

Because of the limited scope of gross pathology, a histological examination was conducted. As shown in *Table 1*, histological analysis of all sections of LSPVs and RSPVs confirmed the presence of ablation lesions (*Figure 5B–D*). However, in the chronic cohort, 47 out of 48 (97.9%) sections achieved transmural lesions. The mean lesion thickness was 0.82 ± 0.54 mm in LSPVs and 1.32 ± 0.60 mm in RSPVs. Only one LSPV did not achieve transmural lesion.

All lesions caused by PFA were continuous, homogenous, and endocardial sparing, with the structural framework preserved in all samples (*Figure 5B–F*). The myocardium was replaced by fibrosis within the lesion. Neither the arteries, veins, nor nerves within the lesion were damaged (*Figure 5C* and *E*). Endocardial oedema was present in acute histopathology (*Figure 5F*) but disappeared in histological sections after 30 days. Haemorrhage and myocardium inflammation were observed in several cases (*Figure 5B*).

No serious IRE-induced complications, such as thrombus, were observed in any of the samples. All the results of the histopathology are summarized in *Table 1*.

## Discussion

In this study, we assessed the feasibility and safety of a lotos PFA catheter as well as a proprietary PFA system designed to achieve ‘single-shot’ PVI for AF. The main findings can be summed up as follows. (i) The catheter was successfully delivered to and contacted all target veins. The procedural workflows were simple and time-saving with a short learning curve for operators. (ii) Pulmonary vein isolation was achieved with a 100% instant success rate in both cohorts. The 30-day isolation rate was 91.6%, indicating excellent effectiveness of the study catheter as well as the PFA system. (iii) No instances of PV stenosis, oesophagus injury, coronary spasm, or microbubbles were observed during and after the procedures, suggesting a level of acceptable safety of the study catheter. (iv) Phrenic nerve capture was observed in 17.6% of applications during ablation with no phrenic palsy in the follow-up period, indicating transient capture rather than sustained injury. (v) In histopathology, the lesions were transmural, continuous, homogenous, and collateral tissue-sparing, with the preservation of the structural framework of the heart. Together, these findings demonstrate that the study catheter and the PFA system are effective and safe for PVI, showing great potential for future clinical applications.

### Catheter design and workflow

This catheter configuration and nanosecond electric pulse setting play a significant role in the effectiveness and safety of ablation. According to previous literature, energy delivery depends on a variety of energy parameters, including pulsed amplitude (voltage), pulse duration, number of pulses, and pulse repetition rate.^17,23,24^ It has been reported that efficacy and tissue damage are dose-dependent,^25,26^ highlighting the importance of carefully tailoring PFA parameters to avoid collateral damage.

We acknowledge that the study catheter and the workflow resemble the ones from flower-shaped PFA catheters including Farawave (Farapulse Inc., CA, USA), but there is indeed quite a difference in terms of catheter structure, parameter settings, energy delivery, and manoeuvering procedure. (i) Although the fully deployed catheter tips are both flower-liked, this study catheter owns more splines and each of the two adjacent splines is linked with electrodes. The novel design contributes to a more stable structure, making the catheter reach the targets easily even with sharp turns as well as enabling optimal contact with various PV ostia diameters. (ii) We also employed a biphasic wave, as in other studies,^19–22,27,28^ to achieve effective treatment with a reduced risk of adverse reactions (e.g. an unwanted temperature increase, phrenic nerve stimulation). (iii) The pulses we generate are on a nanosecond scale. This delivery pattern shortens the delivery time and moderates intracellular effects.^29–31^ In addition, although the underlying explanation is unclear, it reduces the likelihood and extent of phrenic nerve stimulation, as we have observed (see Supplementary material online, *Video S2*). (iv) In terms of operation, the novel lotos design eliminates the need for rotation during PFA deliveries, simplifying the workflow.

Other specificities include the material of the catheter tip, which is made of a flexible material that allows for small catheter deformation that can better fit the PVs in real-world applications. Additionally, there are safety apparatuses. Prior to advance into the LA, an impedance and safety test is performed, and a forced fusion mechanism is available to prevent unintended ablations in time.

A single application for each target has been deemed ideal for PFA innovation. Despite this, there are currently no reliable data on the long-term efficacy of PVI with only one application of PFA energy delivery. Recently, Koruth *et al*. reported that isolation was achieved in 2/2 RSPVs but not in SVCs with one application after a 1 week follow-up period. According to our previous small-sample pilot study, 6/6 PVs achieved isolation after 19 days, while 3/4 PVs exhibited durable isolation after 84 days (see Supplementary material online, *Table S2*). However, damage to the PV antrum was relatively small in the long-term remap (see Supplementary material online, *Figure S2*), suggesting that a single application may not meet the clinical requirements. Therefore, the current protocol was chosen for this study. Further studies are required in order to optimize the catheter and parameters.

There has been a longstanding debate regarding whether contact is necessary to achieve effective PFA or whether contact force is important.^26,32–34^ Howard *et al*.^25^ conducted biphasic, bipolar PFA on an isolated porcine heart model and found that the size of the lesions decreased as the distance between the epicardial surface and the electrodes increased.^26,32^ It is important to note that lesions can form in the absence of direct electrode contact with tissue, making PF delivery more flexible than RF or cryoablation. However, a lack of electrode-tissue contact can result in inadequate lesion formation and ineffective ablation.^35^ Interestingly, basic experiments suggest that achieving tissue contact is more important than the force of contact.^34^ Our study indicates that complete contact is required in order to achieve transmural damage.

The updated literature reports that the 34- mm floral catheter tip (FARAWAVE™, Boston Scientific) increased the incidence of post-operative arrhythmias.^36^ Electroanatomic mapping suggested macro-reentry atrial tachyarrhythmia with a critical isthmus at the left atrial posterior wall. In the present study, the catheter tips are available in two sizes, 28 and 31 mm. Neither post-operative arrhythmias nor reconnection of the left atrial posterior wall on electroanatomic mapping were recorded during the follow-up period. However, for the purpose of preventing unintentional roof line block, further research is necessary in order to identify the optimal range of tip sizes for clinical use.

Overall, the proprietary catheter design and optimized pulse parameters allowing ‘single-shot’ PVI meet the requirements of safety and 30-day efficacy.

### Phrenic nerve capture

In our study, we observed several cases of phrenic nerve capture during pulsed electric field discharges (*Table 3*). This event was associated with the catheter position and the pulse parameters, based on our observation. A previous study has shown that the response of the phrenic nerves is dose-dependent and is affected by the proximity of the catheter to the nerves.^25^ It was found in our study that nanosecond-scale parameters resulted in a significant reduction in phrenic nerve stimulation (see Supplementary material online, *Video S2*).

We observed a higher incidence of the event when ablation was performed in the LSPV, which aligns with the location of the phrenic nerve in swine. However, in previous and our research, neither long-term functional damage nor structural damage caused by pulsed electric fields has been verified.^25,37^ Gross pathology and long-term assessment did not indicate nerve damage, even though the electric field strength exceeded the therapeutic setting when performed directly adjacent to the nerve.^37^ Our pathology results also supported this conclusion. It is speculated that the physiological electrical nerve signalling may have been interfered with or intensified by the transient external electrical field, resulting in the overactivation and unanticipated contraction of the diaphragm muscles, with an absence of irreversible damage.

Based on our experimental results, when the catheter was ablated in a spindle shape rather than a lotos shape, phrenic nerve capture occurred more frequently. Possibly, this is due to the smaller electrode spacing in the spindle shape compared with the lotos shape. According to basic research, the delivery energy is negatively correlated with electrode spacing,^24^ which may explain the observation. According to this, real-time electrode spacing calculations and current density estimation based on three-dimensional mapping and computational system may be convenient for operators in practical use and represent an important direction for future research.

### Histopathology in our study

In histopathology, the lesions were transmural, continuous, homogenous, and endocardial sparing, with the preservation of the structural integrity of the heart. Fibrous collagen and inflammation were involved in the process. No thrombus or injury to the arterial, venular, or nerve structures was observed. These findings are consistent with the previously described characteristics of the non-thermal ablation mechanism of PFA.^24,38^

### Limitations

In this study, safety and chronic lesions were demonstrated for a relatively short-term follow-up period (up to 30 days), while long-term follow-up studies, such as those conducted for 3 months, are necessary to provide a comprehensive evaluation of durability and safety.

As to histopathology, there were only four longitudinal sections per targeted vein. Segments that were not ablated or did not have transmural lesions were likely missed, resulting in an overestimation of circumferentiality and transmurality. Additionally, it is noteworthy that acute pathology was performed within 30 min after the procedure when the injury foci were not fully developed. Further studies are required to identify acute inflammatory reactions and other acute pathological changes at 7 days post procedure.

For safety, there may be a need for quantitative studies investigating the factors affecting the capture of the phrenic nerve, which are not included in our study and will be investigated in the future. Furthermore, the impact of PFA on ganglionated plexi (GP) and the cardiac autonomic nervous system (ANS) was not investigated in this study. It has been reported that classic PVI procedures using thermal ablations (RF or CB) have an effect on GPs and the ANS.^39^ However, there is controversy regarding whether additional GP ablation can prevent the recurrence of AF.^40,41^ Recent research has shown that GPs are not adversely affected by PFA during endocardial ablation.^42–44^ Therefore, comparative experiments are necessary to determine whether PFA damages the ANS and whether it impacts the recurrence of AF.

Last but not least, as humans and swine have different anatomical and physiological states, there is a limitation in predicting the efficacy and safety of PFA in humans. Clinical evaluations should be conducted separately and with caution.

## Conclusions

Excellent PVI efficacy and acceptable safety can be achieved by this novel lotos PFA catheter and PFA system. It is capable of creating transmural myocardial lesions, while sparing the coronary arteries and the phrenic nerve. Furthermore, it can isolate the PVs without acute or chronic PV stenosis and oesophagus injury.

## Supplementary Material

euad362_Supplementary_Data

## Data Availability

Data and methods used in the analysis and materials used to conduct the research will not be available for access.

## References

[1] Hindricks G, Potpara T, Dagres N, Arbelo E, Bax JJ, Blomström-Lundqvist C et al 2020 ESC guidelines for the diagnosis and management of atrial fibrillation developed in collaboration with the European Association for Cardio-Thoracic Surgery (EACTS): the task force for the diagnosis and management of atrial fibrillation of the European Society of Cardiology (ESC) developed with the special contribution of the European Heart Rhythm Association (EHRA) of the ESC. Eur Heart J 2021;42:373–498. 32860505 10.1093/eurheartj/ehaa612

[2] Haïssaguerre M, Jaïs P, Shah DC, Takahashi A, Hocini M, Quiniou G et al Spontaneous initiation of atrial fibrillation by ectopic beats originating in the pulmonary veins. N Engl J Med 1998;339:659–66. 9725923 10.1056/NEJM199809033391003

[3] Haïssaguerre M, Jaïs P, Shah DC, Garrigue S, Takahashi A, Lavergne T et al Electrophysiological end point for catheter ablation of atrial fibrillation initiated from multiple pulmonary venous foci. Circulation 2000;101:1409–17. 10736285 10.1161/01.cir.101.12.1409

[4] Verma A, Jiang C, Betts TR, Chen J, Deisenhofer I, Mantovan R et al Approaches to catheter ablation for persistent atrial fibrillation. N Engl J Med 2015;372:1812–22. 25946280 10.1056/NEJMoa1408288

[5] Kuck KH, Brugada J, Fürnkranz A, Metzner A, Ouyang F, Chun KRJ et al Cryoballoon or radiofrequency ablation for paroxysmal atrial fibrillation. N Engl J Med 2016;374:2235–45. 27042964 10.1056/NEJMoa1602014

[6] van Driel VJ, Neven KG, van Wessel H, du Pré BC, Vink A, Doevendans PA et al Pulmonary vein stenosis after catheter ablation: electroporation versus radiofrequency. Circ Arrhythm Electrophysiol 2014;7:734–8. 24958397 10.1161/CIRCEP.113.001111

[7] Kuroki K, Whang W, Eggert C, Lam J, Leavitt J, Kawamura I et al Ostial dimensional changes after pulmonary vein isolation: pulsed field ablation vs radiofrequency ablation. Heart Rhythm 2020;17:1528–35. 32380290 10.1016/j.hrthm.2020.04.040

[8] Lemoine MD, Mencke C, Nies M, Obergassel J, Scherschel K, Wieboldt H et al Pulmonary vein isolation by pulsed-field ablation induces less neurocardiac damage than cryoballoon ablation. Circ Arrhythm Electrophysiol 2023;16:e011598. 36938715 10.1161/CIRCEP.122.011598

[9] Reddy VY, Dukkipati SR, Neuzil P, Anic A, Petru J, Funasako M et al Pulsed field ablation of paroxysmal atrial fibrillation: 1-year outcomes of IMPULSE, PEFCAT, and PEFCAT II. JACC Clin Electrophysiol 2021;7:614–27. 33933412 10.1016/j.jacep.2021.02.014

[10] Musikantow DR, Neuzil P, Anic A, Balin P, Petru J, Funasako M et al Long-term clinical outcomes of pulsed field ablation in the treatment of paroxysmal atrial fibrillation. JACC Clin Electrophysiol 2023;9:2001–3. 37565951 10.1016/j.jacep.2023.06.019

[11] Reddy VY, Anic A, Koruth J, Petru J, Funasako M, Minami K et al Pulsed field ablation in patients with persistent atrial fibrillation. J Am Coll Cardiol 2020;76:1068–80. 32854842 10.1016/j.jacc.2020.07.007

[12] Turagam MK, Neuzil P, Schmidt B, Reichlin T, Neven K, Metzner A et al Safety and effectiveness of pulsed field ablation to treat atrial fibrillation: one-year outcomes from the MANIFEST-PF registry. Circulation 2023;148:35–46. 37199171 10.1161/CIRCULATIONAHA.123.064959

[13] Verma A, Haines DE, Boersma LV, Sood N, Natale A, Marchlinski FE et al Pulsed field ablation for the treatment of atrial fibrillation: PULSED AF pivotal trial. Circulation 2023;147:1422–32. 36877118 10.1161/CIRCULATIONAHA.123.063988 PMC10158608

[14] Reddy VY, Gerstenfeld EP, Natale A, Whang W, Cuoco FA, Patel C et al Pulsed field or conventional thermal ablation for paroxysmal atrial fibrillation. N Engl J Med 2023;389:1660–71. 37634148 10.1056/NEJMoa2307291

[15] Kaminska I, Kotulska M, Stecka A, Saczko J, Drag-Zalesinska M, Wysocka T et al Electroporation-induced changes in normal immature rat myoblasts (H9C2). Gen Physiol Biophys 2012;31:19–25. 22447827 10.4149/gpb_2012_003

[16] Avazzadeh S, O’Brien B, Coffey K, O’Halloran M, Keane D, Quinlan LR. Establishing irreversible electroporation electric field potential threshold in a suspension in vitro model for cardiac and neuronal cells. J Clin Med 2021;10:5443. 34830725 10.3390/jcm10225443 PMC8622402

[17] Tabaja C, Younis A, Hussein AA, Taigen TL, Nakagawa H, Saliba WI et al Catheter-based electroporation: a novel technique for catheter ablation of cardiac arrhythmias. JACC Clin Electrophysiol 2023;9:2008–23. 37354168 10.1016/j.jacep.2023.03.014

[18] Wintgens LIS, Klaver MN, Maarse M, Spitzer SG, Langbein A, Swaans MJ et al Efficacy and safety of the GOLD FORCE multicentre randomized clinical trial: multielectrode phased radiofrequency vs. irrigated radiofrequency single-tip catheter with contact force ablation for treatment of symptomatic paroxysmal atrial fibrillation. Europace 2021;23:1931–8. 34279627 10.1093/europace/euab168

[19] Koruth J, Kuroki K, Iwasawa J, Enomoto Y, Viswanathan R, Brose R et al Preclinical evaluation of pulsed field ablation: electrophysiological and histological assessment of thoracic vein isolation. Circ Arrhythm Electrophysiol 2019;12:e007781. 31826647 10.1161/CIRCEP.119.007781 PMC6924932

[20] Stewart MT, Haines DE, Verma A, Kirchhof N, Barka N, Grassl E et al Intracardiac pulsed field ablation: proof of feasibility in a chronic porcine model. Heart Rhythm 2019;16:754–64. 30385383 10.1016/j.hrthm.2018.10.030

[21] Koruth J, Kawamura I, Dukkipati SR, Neuzil P, Reddy VY. Preclinical assessment of the feasibility, safety and lesion durability of a novel ‘single-shot’ pulsed field ablation catheter for pulmonary vein isolation. Europace 2023;25:1369–78. 36794699 10.1093/europace/euad030 PMC10105880

[22] Hsu JC, Gibson D, Banker R, Doshi SK, Gidney B, Gomez T et al In vivo porcine characterization of atrial lesion safety and efficacy utilizing a circular pulsed-field ablation catheter including assessment of collateral damage to adjacent tissue in supratherapeutic ablation applications. J Cardiovasc Electrophysiol 2022;33:1480–8. 35510408 10.1111/jce.15522 PMC9545022

[23] Maor E, Sugrue A, Witt C, Vaidya VR, DeSimone CV, Asirvatham SJ et al Pulsed electric fields for cardiac ablation and beyond: a state-of-the-art review. Heart Rhythm 2019;16:1112–20. 30641148 10.1016/j.hrthm.2019.01.012

[24] Di Biase L, Diaz JC, Zhang XD, Romero J. Pulsed field catheter ablation in atrial fibrillation. Trends Cardiovasc Med 2022;32:378–87. 34329732 10.1016/j.tcm.2021.07.006

[25] Howard B, Haines DE, Verma A, Kirchhof N, Barka N, Onal B et al Characterization of phrenic nerve response to pulsed field ablation. Circ Arrhythm Electrophysiol 2022;15:e010127. 35649121 10.1161/CIRCEP.121.010127 PMC9213085

[26] Meckes D, Emami M, Fong I, Lau DH, Sanders P. Pulsed-field ablation: computational modeling of electric fields for lesion depth analysis. Heart Rhythm O2 2022;3:433–40. 36097449 10.1016/j.hroo.2022.05.009 PMC9463712

[27] Koruth J, Verma A, Kawamura I, Reinders D, Andrade JG, Deyell MW et al PV isolation using a spherical array PFA catheter: preclinical assessment and comparison to radiofrequency ablation. JACC Clin Electrophysiol 2023;9:652–66. 36842871 10.1016/j.jacep.2023.01.022

[28] Yavin H, Brem E, Zilberman I, Shapira-Daniels A, Datta K, Govari A et al Circular multielectrode pulsed field ablation catheter lasso pulsed field ablation. Circ Arrhythm Electrophysiol 2021;14:e009229. 33417475 10.1161/CIRCEP.120.009229 PMC7909749

[29] Napotnik TB, Reberšek M, Vernier PT, Mali B, Miklavčič D. Effects of high voltage nanosecond electric pulses on eukaryotic cells (in vitro): a systematic review. Bioelectrochemistry 2016;110:1–12. 26946156 10.1016/j.bioelechem.2016.02.011

[30] Asadipour K, Hani MB, Potter L, Ruedlinger BL, Lai N, Beebe SJ. Nanosecond pulsed electric fields (nsPEFs) modulate electron transport in the plasma membrane and the mitochondria. Bioelectrochemistry 2023;155:108568. 37738861 10.1016/j.bioelechem.2023.108568

[31] Rajagopalan NR, Munawar T, Sheehan MC, Fujimori M, Vista WR, Wimmer T et al Electrolysis products, reactive oxygen species and ATP loss contribute to cell death following irreversible electroporation with microsecond-long pulsed electric fields. Bioelectrochemistry 2023;155:108579. 37769509 10.1016/j.bioelechem.2023.108579 PMC10841515

[32] Howard B, Verma A, Tzou WS, Mattison L, Kos B, Miklavčič D et al Effects of electrode-tissue proximity on cardiac lesion formation using pulsed field ablation. Circ Arrhythm Electrophysiol 2022;15:e011110. 36166690 10.1161/CIRCEP.122.011110 PMC9584049

[33] Hocini M, Condie C, Stewart MT, Kirchhof N, Foell JD. Predictability of lesion durability for AF ablation using phased radiofrequency: power, temperature, and duration impact creation of transmural lesions. Heart Rhythm 2016;13:1521–6. 26921762 10.1016/j.hrthm.2016.02.012

[34] Mattison L, Verma A, Tarakji KG, Reichlin T, Hindricks G, Sack KL et al Effect of contact force on pulsed field ablation lesions in porcine cardiac tissue. J Cardiovasc Electrophysiol 2023;34:693–9. 36640426 10.1111/jce.15813

[35] Gasperetti A, Assis F, Tripathi H, Suzuki M, Gonuguntla A, Shah R et al Determinants of acute irreversible electroporation lesion characteristics after pulsed field ablation: the role of voltage, contact, and adipose interference. Europace 2023;25:euad257. 37649337 10.1093/europace/euad257 PMC10485186

[36] Tohoku S, Chun KRJ, Bordignon S, Chen S, Schaack D, Urbanek L et al Findings from repeat ablation using high-density mapping after pulmonary vein isolation with pulsed field ablation. Europace 2023;25:433–40. 36427201 10.1093/europace/euac211 PMC9935020

[37] van Driel VJ, Neven K, van Wessel H, Vink A, Doevendans PA, Wittkampf FH. Low vulnerability of the right phrenic nerve to electroporation ablation. Heart Rhythm 2015;12:1838–44. 25998897 10.1016/j.hrthm.2015.05.012

[38] Grimaldi M, Di Monaco A, Gomez T, Berman D, Datta K, Sharma T et al Time course of irreversible electroporation lesion development through short- and long-term follow-up in pulsed-field ablation–treated hearts. Circ Arrhythm Electrophysiol 2022;15:e010661. 35763432 10.1161/CIRCEP.121.010661

[39] Verma A, Saliba WI, Lakkireddy D, Burkhardt JD, Cummings JE, Wazni OM et al Vagal responses induced by endocardial left atrial autonomic ganglion stimulation before and after pulmonary vein antrum isolation for atrial fibrillation. Heart Rhythm 2007;4:1177–82. 17765618 10.1016/j.hrthm.2007.04.023

[40] Katritsis DG, Pokushalov E, Romanov A, Giazitzoglou E, Siontis GC, Po SS et al Autonomic denervation added to pulmonary vein isolation for paroxysmal atrial fibrillation: a randomized clinical trial. J Am Coll Cardiol 2013;62:2318–25. 23973694 10.1016/j.jacc.2013.06.053

[41] Driessen AHG, Berger WR, Krul SPJ, van den Berg NWE, Neefs J, Piersma FR et al Ganglion plexus ablation in advanced atrial fibrillation: the AFACT study. J Am Coll Cardiol 2016;68:1155–65. 27609676 10.1016/j.jacc.2016.06.036

[42] Musikantow DR, Neuzil P, Petru J, Koruth JS, Kralovec S, Miller MA et al Pulsed field ablation to treat atrial fibrillation: autonomic nervous system effects. JACC Clin Electrophysiol 2023;9:481–93. 36752473 10.1016/j.jacep.2022.10.028

[43] Tohoku S, Schmidt B, Schaack D, Bordignon S, Hirokami J, Chen S et al Impact of pulsed-field ablation on intrinsic cardiac autonomic nervous system after pulmonary vein isolation. JACC Clin Electrophysiol 2023;9:1864–75. 37480870 10.1016/j.jacep.2023.05.035

[44] O’Brien B, Reilly J, Coffey K, González-Suárez A, Quinlan L, van Zyl M. Cardioneuroablation using epicardial pulsed field ablation for the treatment of atrial fibrillation. J Cardiovasc Dev Dis 2023;10:238. 37367403 10.3390/jcdd10060238 PMC10299113

